# Smartphone-based 3D facial scanning applications in orthodontic diagnosis: A cross-sectional study

**DOI:** 10.34172/joddd.025.42109

**Published:** 2025-06-30

**Authors:** Amritha Nehru, Shweta Nagesh

**Affiliations:** Department of Orthodontics, Saveetha Dental College and Hospitals, Saveetha Institute of Medical and Technical Sciences, Saveetha University, Chennai, India

**Keywords:** Face, Mobile applications, Orthodontics, Soft tissues, Smartphone, Three-dimensional imaging

## Abstract

**Background.:**

Innovations in smartphone technology have transformed diagnostics in healthcare. In orthodontics, these applications can serve as potential diagnostic tools. The present study analyzed the accuracy of two smartphone-based face scanners compared to a standard CBCT-based facial scanner and evaluated scanning duration, user experience, and preferences for orthodontic diagnostics.

**Methods.:**

This cross-sectional study included 15 individuals aged 18–25 years. Each participant was scanned with a CBCT-based scanner (group 1), SureScan 3D App (group 2), and QLone App (group 3). Accuracy was assessed by superimposing scans using MeshLab and Geomagic Control X software. Scanning duration was recorded. Additionally, 30 orthodontists completed a questionnaire to evaluate user experience with face scans. ANOVA and Kruskal-Wallis tests compared the accuracy and scanning duration, respectively, while the Mann-Whitney U test was used to assess region-based reproducibility. *P*≤0.05 was considered significant.

**Results.:**

Smartphone-based apps showed overall accuracy comparable to the conventional scanner (*P*=0.765). SureScan 3D had the lowest mean error (1.53±0.24 mm). In region-based superimposition, the cheeks and forehead had high reproducibility, followed by the nose with moderate and the perioral area with poor reproducibility. Significant differences in scanning duration were observed (*P*=0.001), with SureScan 3D having the shortest scanning time (52.80±4.17 seconds) and 93.3% of orthodontists viewed facial scans as a substitute for photographs, though 53.3% still preferred photographs.

**Conclusion.:**

Smartphone face scanning applications offer accuracy comparable to standard scanners with reduced scanning times. They provide a cost-efficient and reliable alternative to traditional scanners for orthodontic documentation.

## Introduction

 The ongoing growth in digital technology has resulted in significant advancements in dentistry and treatment workflow. These advancements have various applications in diagnosis, treatment planning, assessment of treatment changes, and patient communication.^1,2^ This also applies to the transition towards a fully digital workflow in orthodontics, encompassing digital diagnostics, novel materials, and customized appliances. This procedure is not new, having started several decades ago with the advent of digital x-ray devices and, subsequently, CBCT imaging. In addition, the advent of clear aligners, intraoral scanners, and associated digital treatment planning catalyzed the development of a 3D workflow in orthodontics.^3^ Digital technology in three dimensions has transformed orthodontic diagnosis, extending beyond hard tissue imaging. Advances in soft tissue imaging have made it possible to visualize facial soft tissues in three dimensions using face scanning technologies. Currently available face scanners are based on four principles: photogrammetry, stereophotogrammetry, structured light scanning, and laser scanning.^4^ All these methods are accurate and reproducible. These scanners have the potential to replace conventional extraoral records. However, some of the common disadvantages of the standard face scanners are the lack of portability, high cost, and the large size that hinder widespread clinical use.^5,6^

 To address these limitations, research by Elbashti et al^7^ and Amornvit and Environ^8^ advocated using smartphones as an alternative to photogrammetry for capturing facial soft tissues. A smartphone can function as a cost-effective substitute for acquiring scans with exceptional accuracy. Newer smartphone versions, using internal cameras and structured infrared light, can generate high-quality 3D scans.^9^ Their success is attributed to the TrueDepth camera and Light Detection and Ranging technology (LiDAR). The integrated TrueDepth camera uses a light-emitting diode to generate a grid of over 30 000 infrared dots, enabling the capture of depth in milliseconds.^9^ The TrueDepth scanning done on an iPhone or iPad with a 3D capture device can be an economical alternative for facial scanning. Using these technologies, various mobile applications are available currently, like 3DFaceScan, QLone, Polycam 3D, EM3D, Heges 3D, Scandypro, 3DsizeME, SureScan, Magiscan, and Scaniverse. Most of these applications are compatible with an iPhone 12 or later, while few are available with Android. Of these applications, the most economical was the QLone (EyeCue Vision Technologies Ltd).^10^ The QLone works on the principle of photogrammetry without the need for LiDAR sensors.^10^ Applications, such as SureScan and Polycam, use the TrueDepth camera and LiDAR sensors. They also provide economically viable in-app purchases. Nonetheless, Polycam has been previously examined in several studies; thus, we incorporated QLone and SureScan into the current investigation due to the limited research validating their accuracy.^9,10^

 Despite these advancements, not all smartphone-based applications were developed exclusively for use in dentistry. Hence, it is crucial to validate the accuracy of the scans compared to standard facial scanners to determine their suitability for clinical use. Previous studies by Thurzo et al^11^ and Pellitteri et al^12,13^ evaluated the Bellus 3D Application against CBCT-based facial scanners and structured light-based scanners. However, the Bellus 3D program is no longer available. Therefore, it is essential to evaluate the precision of the existing facial scanning applications for possible orthodontic diagnostic use. Also, the operator’s experience and willingness to adapt to the new technology have not been evaluated previously. The primary objective of this study was to assess the accuracy and scanning time of two smartphone-based facial scanning applications against a standard CBCT-based facial scanner. The second objective was to evaluate the user experience and preference regarding face scanners compared to conventional photographs for orthodontic diagnosis.

## Methods

###  Study design and participants

 The present cross-sectional study was conducted following approval from the institutional ethics committee (SRB/SDC/ORTHO-2301/23/207) and obtaining informed consent from all participants. Fifteen subjects (8 men and 7 women) were recruited, meeting the following inclusion and exclusion criteria. The inclusion criteria were non-growing patients aged 18‒25 years. The exclusion criteria were patients with facial deformities, patients with severe skeletal malocclusion and asymmetry, a history of facial trauma and scarring, those who underwent facial plastic surgery, or, in the case of men, those who had beards that could obstruct the scanning process.

###  Sample size and study procedures

 G*Power software version 3.1 (Franz Faul, University of Kiel, Germany) was used to determine the sample size based on a previous study by Pellitteri et al.^13^ For an estimated effect size of 21.25 from the previous study, with a power of 95% and an α error of 0.05, a minimum of 10 patients were required. The present study included 15 subjects per group and 45 facial scans across three groups: group 1: CBCT-based face scanner (CS face scan kit, CS 9600, Carestream Dental LLC), group 2: SureScan 3D Application (Xyken, LLC), and group 3: Qlone Application (EyeCue Vision Technologies Ltd, Israel). Table 1 details the face scanners used in the study. The study enrolled all participants from the same dental hospital where the study was conducted. All the participants were patients who presented to the orthodontic department for treatment. Each participant underwent facial scanning with all three devices on the same day, with scans performed consecutively by a single operator. The scanning duration for each device was recorded using a timer. Before scanning, the participants removed all accessories that might interfere with image capture and secured their hair to fully expose the facial skin, including the forehead and ears.

**Table 1:** Face Scanning Software used in the study

**Specifications**	**CS Face Scanning software**	**SureScan 3D App**	**QLone App**
Manufacturer	Carestream Dental LLC Group	Xyken, LLC	EyeCue Vision Technologies Ltd
Type of scanner	Add on a face scan kit with CBCT imaging	Smartphone-based application	Smartphone-based application
Technology used	Photogrammetry	True depth Scanning technology with LiDAR sensor	Photogrammetry with AI algorithm
Operating system	-	iOS 15.0 or later	iOS 12.0 or later, Android 7.0 or later
Cost	3000 USD	In-app purchase with a monthly or yearly subscription model. Monthly- 24 USD; yearly- up to 300 USD	In-app purchase with unlimited premium features from 14-35 USD
Size	-	13.9 MB	115.5 MB

###  Data acquisition and processing

 All the scans were recorded in a well-illuminated photography room. CS3D face scanner (group 1) was used to capture the first set of scans. All the participants were seated on a height-adjustable stool and instructed to maintain a neutral facial expression and natural head position (NHP) during scanning. The face scanner was equipped with a head positioner to stabilize the participants’ heads. A single, trained faculty member from the radiology department captured all the facial scans. The scans were captured based on the manufacturer’s instructions. The CS facial scan is independent of the CBCT image and does not expose the patients to radiation. For the SureScan 3D App (group 2), the smartphone was mounted on a tripod at a 30-cm distance. The participants were asked to rotate their heads while keeping the NHP, following protocols similar to the procedure described in the study by D’ettorre et al.^14^ The SureScan 3D application uses the front camera for image capture. The QLone App (group 3) required the operator to move the smartphone in a cross pattern around the participant’s face from a 30-cm distance, capturing all facial aspects. The app’s “Clean” tool removed background elements after the scan before exporting the scans. The same investigator conducted both application-based scanning tasks on a single smartphone (iPhone 15, Apple Inc). The iPhone had a 48-megapixel camera with a 12-megapixel TrueDepth camera, an A16 bionic chip with a 5-core GPU, and 8 GB RAM. A single investigator recorded all the scans, exported the images as STL files, and processed them using MeshLab software (Version 2023.12, Italian National Research Council], Rome, Italy) and Geomagic Control X software (Geomagic, Morrisville, USA).

###  Parameters assessed

####  Accuracy

**Figure 1:**
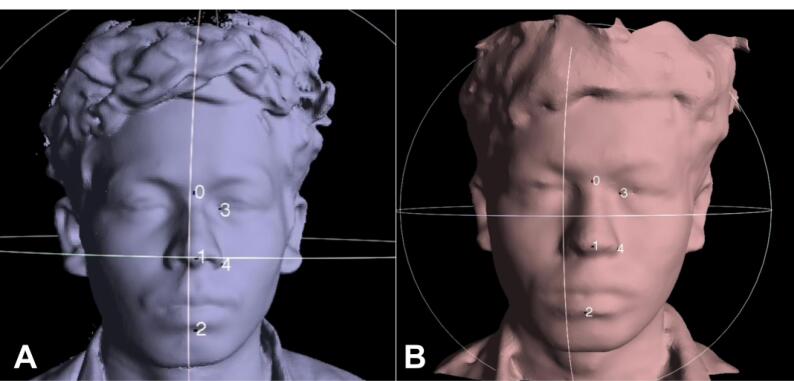
Superimposition of the face scans was done using point-based gluing in the MeshLab software to assess the mean deviation error. A: CS Face Scan; B: SureScan 3D

 The scans were superimposed between the groups in three combinations. The overall accuracy for each combination was evaluated using the MeshLab software by superimposing the scans using point-based gluing, where various points like the glabella, inner and outer canthus of the eye, and commissures of the lip and cheeks were matched based on manufacturer instructions (Figure 1). The accuracy was expressed by mean deviation errors based on a previous study.^12^ The superimposition for one sample was done in triplicate, and a mean value was considered for statistical analysis. Region-based superimposition was done using Geomagic Control X software by superimposing scans of groups 2 and 3 with group 1 and measuring deviations in four specific facial areas (forehead, nose, cheeks, and perioral regions) using color maps. Surface-to-surface differences were shown by the color maps. These differences were matched by tolerance bands: highly reproducible (0.5 mm to 0 mm and 0 mm to −0.5 mm), moderately reproducible (1 mm to 0.5 mm and −0.5 mm to −1 mm), poorly reproducible (1.5 mm to 1 mm and −1 mm to −1.5 mm), and not reproducible ( > 1.5 mm and < −1.5 mm). The percentage of tolerance bands in each superimposition was computed and analyzed.

####  Scanning time

 As the scans were being captured, another investigator recorded the time taken to capture the face scans. The time was calculated from the start of the scanning process until the 3D face scan was generated on the screen.

####  User preference and experience

 Thirty orthodontists, including experienced clinical practitioners and residents, were randomly selected to use the QLone and SureScan 3D applications for patient image capturing. The orthodontists and residents were selected from the same hospital where the study was conducted. Before commencing orthodontic treatment, all participants routinely used a DSLR camera with a ring flash for extraoral patient documentation. They were instructed to use both the face scan app and the photographs for a specific patient. A questionnaire then assessed the user’s experiences and perceptions. The questionnaire was sent out using a Google Form to their email addresses. The questionnaire was adapted from prior research.^15^

###  Statistical analysis

 Data were analyzed using SPSS (IBM, Chicago, IL). The Kolmogorov-Smirnov test evaluated the normality of the data distribution. The repeated-measures ANOVA or equivalent non-parametric tests were used to evaluate the overall scanning accuracy and duration between the three groups. Bonferroni’s post hoc comparisons were used to mitigate type I errors in multiple comparisons. The Mann-Whitney test was used to evaluate the consistency of surface area measurements between smartphone-based scans and CS Face scan imaging. We quantified response frequencies and percentages to assess operator perception. *P* values of ≤ 0.05 were deemed statistically significant.

## Results

 This cross-sectional survey was performed for three months, from May to July 2024. The mean age of the participants was 24.9 ± 4.9 years. The normality tests indicated that the total accuracy scores were normally distributed. Nevertheless, the repeatability percentages and scanning duration depending on regions were not normally distributed. Therefore, non-parametric tests were employed for comparison.

###  Accuracy

 The lowest mean error was seen when comparing groups 1 and 2 (1.53 ± 0.24mm). The mean error for group 1‒group 3 was 1.62 ± 0.38 mm. The mean error on superimposing group 2‒group 3 was 1.62 ± 0.28 mm. ANOVA was used to compare the mean values, which showed no statistically significant differences between the three groups (*P* = 0.765**)**. These findings suggest that the smartphone-based face scanning applications (groups 2 and 3) demonstrated comparable overall accuracy to group 1 (Table 2).

**Table 2:** Means and standard deviations (mm) of the overall accuracy of the superimposed scans

	**Groups**			***P*** ** value**
	**Group 1 vs. group 2**	**Group 1 vs. group 3**	**Group 2 vs. group 3**	
Mean and SD (mm)	1.53 ± 0.24	1.62 ± 0.38	1.62 ± 0.28	0.765
95% Confidence interval	1.36‒1.71	1.35‒1.89	1.42‒1.83	

*Note:*One-way ANOVA was used to assess the differences between the groups.

 The present study used the Mann-Whitney U test (Table 3) to compare the surface-to-surface reproducibility of groups 2 and 3 to group 1 across four facial areas: the cheek, the nose, the forehead, and the area around the mouth. In the cheek area, groups 1‒3 exhibited a larger percentage of high reproducibility ratings (86.7%) compared to groups 1‒2 (66.7%). Groups 1‒2 exhibited moderate reproducibility more frequently (33.3%) than groups 1‒3 (13.3%). Nonetheless, a *P* value of 0.20 indicates that these differences were not statistically significant. Both groups 1‒2 and 1‒3 achieved comparable results in the nasal area, with 20.0% of scans achieving high reproducibility and the remaining scans classified as having moderate reproducibility. Group 2 had a singular occurrence of poor reproducibility (6.7%). However, a *P* value of 0.73 signified a lack of statistical significance. In the forehead area, groups 1‒2 achieved high reproducibility in 66.7% of scans, while groups 1‒3 achieved 80.0%. Groups 1‒2 had a slightly higher prevalence of moderate reproducibility (33.3%) compared to groups 1‒3 (20.0%). The perioral area exhibited a greater incidence of poor reproducibility in groups 1‒3 (60.0%) relative to groups 1‒2 (40.0%), but moderate reproducibility was more prevalent in groups 1‒2 (53.3%) than in groups 1‒3 (33.3%). Both groups showed a lower rate of high reproducibility ratings at 6.7%. The differences observed were not statistically significant (*P* = 0.32). The results demonstrated that although there were variations in the percentages of high, moderate, and low reproducibility ratings between the groups, none of these differences attained statistical significance (Table 3).

**Table 3:** Percentages of accuracy values based on regional superimposition of groups 2 and 3 with group 1

**Outcome**	**Scoring**	**Group 1‒Group 2 No. (%)**	**Group 1‒Group 3 No. (%)**	**Mann-Whitney U test value**	***P*** ** value**
Cheek	High	10 (66.7%)	13 (86.7%)	90.00	0.20
	Moderate	5 (33.3%)	2 (13.3%)		
	Poor	0 (0%)	0 (0%)		
Nose	High	3 (20.0%)	3 (20.0%)	106.50	0.73
	Moderate	11 (73.3%)	12 (80.0%)		
	Poor	1 (6.7%)	0 (0%)		
Forehead	High	10 (66.7%)	12 (80.0%)	97.50	0.41
	Moderate	5 (33.3%)	3 (20.0%)		
	Poor	0 (0%)	0 (0%)		
Perioral	High	1 (6.7%)	1 (6.7%)	91.50	0.32
	Moderate	8 (53.3%)	5 (33.3%)		
	Poor	6 (40.0%)	9 (60.0%)		

*Note: *Mann-Whitney U test was used to compare the accuracy percentages.

###  Scan time

 The comparison of scanning durations between the three groups indicated significant differences (*P* = 0.001) (Table 4). Group 1 had the highest average scanning time (145.73 ± 19.78 seconds), followed by group 3 (115.13 ± 22.00 seconds), whereas group 2 recorded the lowest scanning time (52.80 ± 4.17 seconds). Using Dunn-Bonferroni to compare groups showed a statistically significant difference between group 1 and group 2 (*P* < 0.001) and between group 2 and group 3 (*P* = 0.001). This showed that group 2 did better than the other groups. The comparison between groups 1 and 3 did not reveal a significant difference (*P* = 0.07) (Table 4).

**Table 4:** Means and standard deviations of the scanning times

**Groups**	**Mean±SD (seconds)**	**95% Confidence interval**	***P *** **value**
Group 1	145.73 ± 19.78	134.78‒156.69	0.001*
Group 2	52.80 ± 4.17	50.49‒55.11	
Group 3	115.13 ± 22.00	102.94‒127.32	
**Intergroup comparison**			
**Groups compared**		**Standard statistical test **	***P *** **value**
Group 1	Group 2	-3.56	< 0.001*
Group 1	Group 3	-5.83	0.07
Group 2	Group 3	-2.27	0.001*

*Note:* Kruskal-Wallis test and intergroup comparison using Dunn-Bonferroni tests were made to evaluate the differences between the three groups (* indicates *P* ≤ 0.05).

###  User preferences and experience

 A questionnaire was administered to 30 orthodontists and orthodontic residents to understand the user experience of the smartphone-based face scanners and their overall preference (Table 5). Convenience sampling was used for this parameter. All the 30 participants completed the questionnaire. The questionnaire results revealed a varied preference between facial scans and photographs for orthodontic extraoral records. Photographs garnered marginal favor for defect identification (50%), data transmission efficiency (56.4%), user convenience (53.3%), and reduced chairside duration (46.7%), while facial scans garnered preference for enhanced patient compliance (50%) and monitoring treatment alterations (50%). Both strategies received an identical rating of 46.7% for ease of mastery among beginners. Facial scans (46.7%) slightly surpassed photos (43.3%) in patient education, but 10% of participants expressed uncertainty. Notwithstanding these discrepancies, 93.3% of participants concurred that facial scans provide a viable option for photographs. Nonetheless, when questioned about their overall preferences, 53.3% preferred photographs, while 46.7% chose facial scans.

**Table 5:** Percentages and response frequencies for the user perception questionnaire

**Outcome**	**Scoring**	**Response frequency (%)**	**Response percentage (%)**
Easier to identify a defect	Facial scan	13	43.3
	Photos	15	50
	Not sure	2	6.7
Easier to master as a beginner	Facial scan	14	46.7
	Photos	14	46.7
	Not sure	2	6.6
Less chair-side time	Facial scan	12	40.0
	Photos	14	46.7
	Not sure	4	13.3
Better patient compliance	Facial scan	15	50
	Photos	14	46.7
	Not sure	1	3.3
Ease of data transfer	Facial scan	13	43.3
	Photos	17	56.4
usage convenience	Facial scan	13	43.3
	Photos	16	53.3
	Not sure	1	3.3
Better tool to detect treatment changes	Facial scan	15	50
	Photos	14	46.7
	Not sure	1	3.3
Better tool for patient education	Facial scan	14	46.7
	Photos	13	43.3
	Not sure	3	10.0
Do you think facial scans are a good alternative to photography?	No	2	6.7
	Yes	28	93.3
What is your preference for recording extraoral records for orthodontic treatment?	Facial scan	14	46.7
	Photos	16	53.3

## Discussion

 This study evaluated the accuracy, reproducibility, and scanning duration of two smartphone-based facial scanning applications against a standard facial scanner. During the selection of the scanning app for the study, cost-effectiveness, good image quality, availability, and ease of use were considered. Previous investigations found that Heges 3D, Scandypro, and EM3D showed problems with accurate image recording.^10^ Given that Polycam and Magiscan have been evaluated previously, QLone and SureScan applications were selected. The research revealed comparable overall accuracy across the scanners. The SureScan 3D app exhibited the lowest deviation value. Nightingale et al^16^ conducted research comparing an iPhone-based face scanner with a gold standard structured light scanner, revealing that iPhone scans were accurate to within 1.3 ± 0.3 mm of the reference scan. The accuracy results of the current study align with the same range. Thurzo et al^11^ indicated that differences of up to 1.5 mm are clinically insignificant. When comparing different areas, the cheeks and forehead had a higher percentage of high reproducibility scores, followed by the nose, which showed moderate reproducibility, and the perioral region, which revealed moderate to poor reproducibility. Consistent with the findings of the current investigation, Thurzo et al^11^ and Pellitteri et al^12^ similarly identified the cheek area as exhibiting good reproducibility. Nonetheless, the current investigation identified the forehead region as exhibiting excellent reproducibility, in contrast to the prior studies.^11-13^ Prior investigations^11-13^ have identified the nose region as fairly replicable, consistent with the findings of the current study. A study by D’Ettorre et al^14^ demonstrated high precision values in the cheek, forehead, and chin regions, consistent with the results of the present investigation. They explained that relatively flat regions exhibited more accuracy than regions with significant curvature, such as the eyes, mouth, and lips.^10^ Consequently, the discrepancies in facial morphology across the samples may have influenced the accuracy of the scans. Furthermore, sustaining a neutral facial expression during the scanning procedure is crucial, since variations in facial expression might affect the accuracy of the scan. This is especially true when the patient must move during the SureScan 3D scanning process. Instructing individuals to maintain a neutral face, verifying their expression before the scan, and assessing elements that influence image quality, such as ambient light, may enhance scan accuracy while maximizing the registration area.^13^ Another notable finding was that the facial scans acquired using smartphone applications exhibited lower resolution and a grainy quality compared to those obtained with the CS facial scanner. Yet, a prior study indicated that this did not impact the precision of the scans.^17^ The aforementioned studies have predominantly evaluated the Bellus 3D application as a representation of smartphone-based facial scanning technology. The application is currently not active.

 On comparing the time taken from initial capture to the final output of the processed image, SureScan 3D was significantly faster than the QLone and CS face scanning. The CS face scan took the longest of the three systems to produce the final processed output. The discrepancy might be attributed to the increased quantity of pictures recorded throughout the scanning procedure. However, an extended scan duration may alter facial expressions, resulting in less accuracy. The study also aimed to get insights into clinical experience and preferences for applying these technologies for orthodontic diagnostics, an aspect that has not been investigated before for face scanning applications. The experience of using smartphone-based scanners was evaluated to measure user acceptance and general perception of their desire to shift to a 3D workflow. Although the survey indicated that operators regard this as a feasible alternative to photographs in orthodontic diagnosis, the majority still preferred 2D photographs in clinical practice. We might attribute this phenomenon to comfort bias, given that the participants were using the applications for the first time within the research context.

 Facial soft tissue is essential for aesthetics, a primary objective in contemporary orthodontic treatments, and sometimes a significant incentive for patients seeking orthodontic care.^18,19^ Smartphone-based scanners provide a cost-effective and portable substitute for traditional scanners.

## Limitations

 The current study had certain limitations. Only two applications were evaluated in comparison to a standard facial scanner. Numerous applications are available on the market that require assessment for their usefulness. Additionally, more factors, such as scan completeness, resolution, and repeatability, were not evaluated. Also, since only the accuracy of the scans was analyzed, intra-rater and inter-rater reliability could not be assessed. The evaluation of scan time did not account for user experience, as all participants were using the scanning applications for the first time. Research indicates that user experience with technology significantly influences scan time, warranting additional evaluation.^20^ The study used an iPhone (iOS) for scanning. Future research might be conducted to compare photo quality and image capture between iPhone and Android smartphones and to optimize smartphone-based applications for clinical situations. Nonetheless, the smartphone applications demonstrated satisfactory accuracy, cost-effectiveness, and rather shorter scan durations. Although the applications may not replicate certain complex facial areas with high accuracy, they provide a viable alternative to traditional 2D images for pre-treatment orthodontic documentation and patient education.

## Conclusion

 This study showed that smartphone face scanning applications, specifically SureScan 3D and QLone, possess accuracy similar to conventional CBCT-based facial scanners in orthodontic diagnosis. SureScan 3D achieved the lowest mean deviation error and markedly decreased scanning duration compared to QLone. While user preferences somewhat favored 2D images for specific elements of orthodontic documentation, a significant majority recognized the feasibility of face scans as an alternative. The findings indicate that smartphone-based facial scanning programs can be useful and economical tools for pre-treatment orthodontic documentation and patient education.

## Competing Interests

 The authors do not have any competing interests to declare.

## Ethical Approval

 The study was conducted according to the Declaration of Helsinki, and prior approval from the institutional ethics committee was obtained before the study. The approval number is SRB/SDC/ORTHO-2301/23/207. Informed written patient consent was obtained from all the participants.
